# Incidental discovery of Amyand's hernia in an adult female: A case report

**DOI:** 10.1016/j.ijscr.2021.106003

**Published:** 2021-05-21

**Authors:** Franck Katembo Sikakulya, Sonye Magugu Kiyaka, Robert Masereka, Patrick Onyai, Philip Anyama

**Affiliations:** a Faculty of Clinical Medicine and Dentistry, Department of Surgery, Kampala International University Western Campus, Ishaka-Bushenyi, Uganda; b Faculty of Medicine, Université Catholique du Graben, Butembo, Democratic Republic of the Congo; c Department of Surgery, Jinja Regional Referral Hospital, Jinja, Uganda

**Keywords:** Amyand's hernia, Adult female, Hernia repair, Case report

## Abstract

**Introduction and importance:**

Amyand's hernia is a rare type of inguinal hernia with an incidence of about 0.1% of all inguinal hernias with most in occurring in childhood. It is characterized by the presence of the vermiform appendix within the hernia sac.

**Case presentation:**

We report the case of 40-year-old female who underwent inguino-labial hernia repair with an incidental finding of a normal appendix within the sac; this was not predicted by the pre-operative ultrasound scan.

**Clinical discussion:**

We recommend that a detailed ultrasound scan be done for all patients with an inguinal hernia to help to manage the patient timeously and safely.

**Conclusion:**

We present a rare condition in a 40-year-old female with a right inguinal hernia, an Amyand's hernia.

## Highlights

• We report a case of Amyand's hernia in a 40-year-old female who underwent inguinal hernia repair.
• A normal appendix was found in the sac and reduced without appendicectomy. Hernia repair without mesh was then performed.
• Ultrasound scan is an excellent technique to evaluate the inguinal region but remains operator dependent.

## 1 Introduction

The vermiform appendix found in an inguinal hernia is called an Amyand's hernia [1]. This hernia is a rare type of inguinal hernia (about 1% of all hernias) with an incidence of about 0.1% of all inguinal hernias. They occur commonly in childhood due to the presence of the patent processus vaginalis [2]. It was first described in 1735 by Claudius Amyand while performing an herniorrhaphy on a 11-year-old boy who presented with an inflamed appendix within an inguinal hernia sac [1]. The appendix located in the inguinal hernia sac can either be normal or incarcerated.

We present a case of Amyand's hernia in a 40-year-old female who underwent hernia repair for a non-painful and reducible right inguinal hernia. This case report has been reported in line with the SCARE 2020 criteria [3].

## 2 Case presentation

**Fig. 1:**
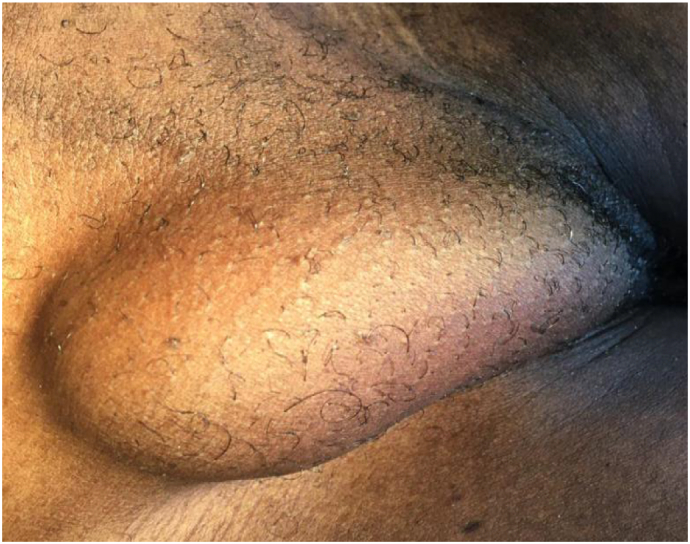
Pre-operative clinical photograph of the right inguinal hernia.

**Fig. 2:**
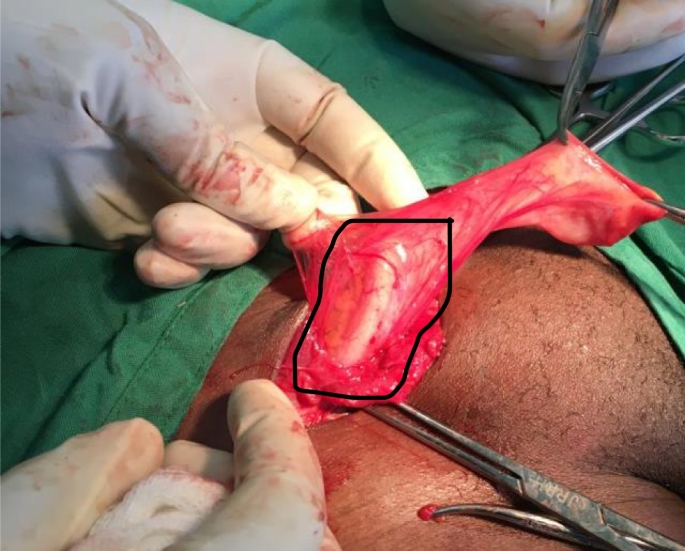
Intra-operative clinical photograph: a normal vermiform appendix within the hernia sac.

**Fig. 3:**
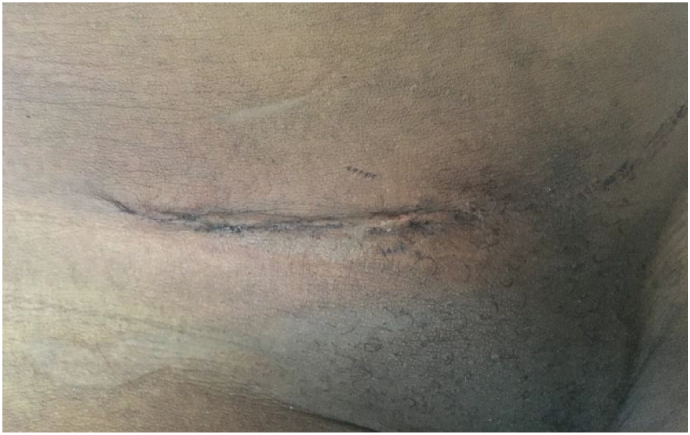
Clinical photograph of the wound on day 2 following surgery.

A 40-year-old female consulted our health facility with a history of a right-sided, non-painful inguinal swelling for a year. The swelling increased in size when coughing or while carrying heavy luggage however it was not associated with constipation or vomiting. On admission, the right inguinal swelling was 3 × 5 cm in diameter and ovoid [Fig. 1]. It had a positive cough impulse, was reducible and non-tender. The deep ring test was positive. An ultrasound scan reported features of right inguinal hernia with a defect of 1.67 cm and without any comments on the contents of the sac. There was no history of previous surgery or any history of chronic disease such as chronic obstructive pulmonary disease or constipation. Routine pre-operative hematological investigations were all within the normal range. The patient denied any allergy to local anesthesia and consented for elective herniorrhaphy under local anesthesia. Surgery was carried out under local anesthesia using 25 ml lidocaine 1% with adrenaline 1:200,000. Incidentally, we found a normal vermiform appendix as the only contents of the sac [Fig. 2]. The appendix was reduced into the peritoneal cavity and a modified Bassini technique was used to repair the hernia. The patient received 2 g of ceftriaxone (50 mg/kg) 30 min prior to the surgery and pain in post-operative period was controlled by 75 mg diclofenac suppository twice daily for three days. The patient reported being satisfied with the technique used and was discharged on day two post-surgery [Fig. 3]. On the 10th post-operative day, she was reviewed and reported no complaints.

## 3 Discussion

Amyand's hernia (AH) is named after Claudius Amyand, who, on December 6, 1735, performed the first successful appendicectomy during the treatment of an 11-year-old boy who presented with a right inguinal hernia. During the surgery, Amyand found a encrusted pin within the appendix and the appendix was found within the sac of the inguinal hernia [1]. Because of the scarcity of the hernia, the term “Amyand's hernia” has recently been adopted as an eponymous description of an appendix within an inguinal hernia [4]. Although Amyand's hernia has been reported in patients ranging in age from 3 weeks to 92 years [5,6], it is 3 times more likely to be diagnosed in children than in adults, due to the patent processus vaginalis [2]. In this case report, we present a 40-year-old female with Amyand's hernia, a condition that is rare in adulthood, especially in females. Its incidence reportedly varies from 0.19% to 1.7% of hernia cases and about 0.1% of all inguinal hernias [2].

The appendix may remain perfectly normal but could become inflamed with subsequent perforation and abscess if the diagnosis is delayed [7]. Pre-operative diagnosis of Amyand's hernia is not straightforward, and is generally an incidental finding during surgery [8]. Physical examination and investigations such as hematological testing and imaging are not always helpful in making the diagnosis [9]. However, it has been reported that ultrasound (USS) is an excellent technique to evaluate the inguinal region and can be used to detect all types of inguinal hernias, including Amyand's hernia [2]. In our case the ultrasound scan did not report the presence of the vermiform appendix and it was instead an incidental finding during hernia repair. The demonstrates the operator dependent nature of USS. Computed tomography (CT) can be used to confirm potential intra-abdominal complications such as perforation and abscess in the pre-operative setting [2,7].

In 2007, Losanoff and Basson described an AH classification and its operative approach, the methods of dealing with the appendix and the type of the hernia repair in adults (Table 1) [10].

**Table 1:** Losanoff–Basson classification of Amyand's hernia (AH) and their management [9].

Types of AH	Features	Surgical management
Type 1	Normal appendix within the inguinal hernia	Reduction of appendix or appendicectomy and mesh hernioplasty
Type 2	Acute appendicitis with no abdominal sepsis	Appendicectomy through the hernia and sutured hernioplasty
Type 3	Acute appendicitis with abdominal sepsis	Appendicectomy through laparotomy with sutured hernioplasty
Type 4	Acute appendicitis associated with related or unrelated abdominal pathology	Appendicectomy through hernia or laparotomy plus diagnostic workup

We report a case of AH without signs of appendicitis and in whom a normal appendix was found; we performed herniorrhaphy without appendicectomy under local anesthesia based on the choice of the patient and the type of anesthesia. The patient recovered well without any complications. Debate continues regarding how to deal with the normal appendix [11] but some authors believe that the normal-looking appendix without any signs of inflammation which is incidentally discovered during surgery should not be removed. They conclude that prophylactic appendicectomy is not necessary [[11], [12]] and that the unnecessary appendicectomy can lead to more post-operative complications.

The review of Desai et al. showed that most surgeons accept the notion of preserving the appendix if it is normal [13]. Some authors have recommended mesh repair for AH with a normal appendix while others have suggested that mesh might increase the chance of wound infection, sepsis and fistula formation [2].

## 4 Conclusion

This presentation reports a case of a rare form of inguinal hernia, Amyand's hernia, in a 40-year-old female. She underwent a hernia repair without appendicectomy as the appendix was found to be normal during surgery. The patient improved and is healthy one month following surgery. Physical examination, laboratory investigations and imaging did not help to preoperative diagnose this incidental Amyand's hernia.

## Sources of funding

There was no external funding source for this report.

## Ethical approval

Not applicable.

## Consent for publication

Written informed consent was obtained from the patient for publication of this case report and accompanying images. A copy of the written consent is available for review by the Editor-in-Chief of this journal on request.

## Author contribution

FKS managed the patient and wrote the first draft. SMK, RM, PO and PA helped in editing and reviewing the paper. All authors read and approved the final version to be published.

## Research registration

Not applicable.

## Guarantor

Franck Katembo Sikakulya.

## Provenance and peer review

Not commissioned, externally peer-reviewed.

## Declaration of competing interest

The authors declare no conflicts of interest.
